# Uncertain impacts on economic growth when stabilizing global temperatures at 1.5°C or 2°C warming

**DOI:** 10.1098/rsta.2016.0460

**Published:** 2018-04-02

**Authors:** Felix Pretis, Moritz Schwarz, Kevin Tang, Karsten Haustein, Myles R. Allen

**Affiliations:** 1 Department of Economics, University of Oxford, Manor Road, Oxford OX1 3UQ, UK; 2 Environmental Change Institute, School of Geography and the Environment, University of Oxford, South Parks Road, Oxford OX1 3QY, UK; 3 Faculty of History, University of Oxford, George Street, Oxford OX1 2BE, UK; 4 Department of Physics, University of Oxford, Parks Road, Oxford OX1 3PU, UK; 5 Programme for Economic Modelling, Institute for New Economic Thinking, Oxford Martin School, University of Oxford, Walton Well Road, Oxford OX2 6ED, UK

**Keywords:** climate, impacts, economic growth, 1.5°C warming scenario, uncertainty, inequality

## Abstract

Empirical evidence suggests that variations in climate affect economic growth across countries over time. However, little is known about the relative impacts of climate change on economic outcomes when global mean surface temperature (GMST) is stabilized at 1.5°C or 2°C warming relative to pre-industrial levels. Here we use a new set of climate simulations under 1.5°C and 2°C warming from the ‘Half a degree Additional warming, Prognosis and Projected Impacts' (HAPPI) project to assess changes in economic growth using empirical estimates of climate impacts in a global panel dataset. Panel estimation results that are robust to outliers and breaks suggest that within-year variability of monthly temperatures and precipitation has little effect on economic growth beyond global nonlinear temperature effects. While expected temperature changes under a GMST increase of 1.5°C lead to proportionally higher warming in the Northern Hemisphere, the projected impact on economic growth is larger in the Tropics and Southern Hemisphere. Accounting for econometric estimation and climate uncertainty, the projected impacts on economic growth of 1.5°C warming are close to indistinguishable from current climate conditions, while 2°C warming suggests statistically lower economic growth for a large set of countries (median projected annual growth up to 2% lower). Level projections of gross domestic product (GDP) *per capita* exhibit high uncertainties, with median projected global average GDP *per capita* approximately 5% lower at the end of the century under 2°C warming relative to 1.5°C. The correlation between climate-induced reductions in *per capita* GDP growth and national income levels is significant at the *p* < 0.001 level, with lower-income countries experiencing greater losses, which may increase economic inequality between countries and is relevant to discussions of loss and damage under the United Nations Framework Convention on Climate Change.

This article is part of the theme issue ‘The Paris Agreement: understanding the physical and social challenges for a warming world of 1.5°C above pre-industrial levels'.

## 1. Introduction

The Paris Agreement proposed to pursue efforts to stabilize global mean surface temperature (GMST) at 1.5°C warming relative to pre-industrial levels. While empirical evidence suggests that climate change affects economic growth across countries and time, studies on the economic impacts of different warming scenarios, including 1.5°C, are scarce. Here we use a new set of climate simulations specified to lead to 1.5°C and 2°C warming relative to pre-industrial levels to project changes in economic growth using empirical estimates of climate impacts.

Economic impacts of climate change have predominantly been studied using integrated assessment models (IAMs) with common examples including DICE [1], FUND [2] and PAGE [3,4]. However, these are rarely tested against observations and are sensitive to the choice of damage or impact functions. Recent doubts about IAMs (see e.g. [5–7] or [8] for a review) have encouraged research to turn towards empirically estimating the impacts of changes in surface temperature and precipitation on socio-economic outcomes (see [9,10] for overviews). Evidence presented therein suggests significant precipitation effects on agricultural outcomes, and nonlinear impacts of temperatures on economic growth across countries (see e.g. Burke, Hsiang & Miguel (BHM) [11]; Dell, Jones & Olken (DJO) [12]; and Sterner [13]).

While climatologists generally agree on a spatially differentiated warming pattern [14], it is not obvious for economic impact projections whether existing climate models could be used directly to project outcomes under 1.5°C (2°C) warming by, for example, shifting a country's temperature down (up) 0.5°C relative to a 2°C (1.5°C) scenario (or up 0.6°C relative to a present-level scenario, which has already seen 0.9°C warming relative to pre-industrial levels), as the spatial distribution of warming under 1.5°C may differ. To compare outcomes specifically under 1.5°C relative to alternative scenarios, we make use of climate simulations from the ‘Half a degree Additional warming, Prognosis and Projected Impacts' (HAPPI) experiment [15]. We obtain simulated climate outcomes on temperature and precipitation under GMST warming of 1.5°C (0.6°C above the 2006–2015 average) and 2°C (1.1°C above the 2006–2015 average). HAPPI provides a large ensemble of simulated possible climate variables under pre-specified warming conditions, allowing us to quantify uncertainties in economic outcomes beyond parameter-estimation uncertainty in impact models.

Most cross-country panel estimation studies of climate impacts rely solely on levels of precipitation and temperatures as explanatory variables, even though more localized evidence suggests that a change in frequency and intensity of climate extremes, together with changes in exposure, affect socio-economic outcomes: from summer heat waves to prolonged cold spells, as well as higher temperature and precipitation variability increasing uncertainty [16]. We thus expand our assessment by testing whether within-year monthly variability measurably impacts annual economic growth beyond nonlinear temperature effects. Additionally, we consider robust estimates of climate impacts by controlling for outlying observations and instabilities not accounted for in estimated models using indicator saturation [17–20].

To assess climate impacts when GMST is stabilized at 1.5°C, we combine HAPPI projections with our econometric estimates of climate impacts. We compare projected impacts on economic growth under no additional warming, 1.5°C and 2°C HAPPI scenarios. These growth impacts are projected without having to specify when the target temperatures of 1.5°C (or 2°C) are reached. We further consider projections of impacts on the levels of gross domestic product (GDP) *per capita* relative to baseline economic scenarios from the shared socio-economic pathways (SSP) [21], assuming the target temperatures are reached at the end of the century.

Panel estimation results that are robust to outliers and breaks suggest that within-year variability of monthly temperatures and precipitation has little measurable effect on annual economic growth beyond global nonlinear temperature effects across countries. While projected temperature changes under a GMST increase of 1.5°C and 2°C lead to proportionally higher warming in the Northern Hemisphere, the projected impact on economic growth is most significant in the Tropics and Southern Hemisphere. Taking into account both econometric estimation and climate uncertainty, the projected impacts on economic growth of 1.5°C warming are close to indistinguishable from current climate conditions, while a 2°C warming suggests statistically lower economic growth for a large set of countries, with median projections of a reduction in annual growth of up to 2%. The correlation between additional reductions in *per capita* GDP growth between 1.5°C and 2.0°C and national income levels is significant at the *p* < 0.001 level, with lower-income countries experiencing greater reductions, which may lead to increased economic inequality across countries and is relevant to discussions of loss and damage under the United Nations Framework Convention on Climate Change (UNFCCC). Projections of climate impacts on the levels of GDP *per capita* using SSP scenarios exhibit high uncertainties, with median projected global GDP *per capita* being approximately 5% lower at the end of the century under 2°C warming relative to 1.5°C. Relative to a scenario without additional warming, global average GDP *per capita* is estimated to be 8% lower under 1.5°C warming and 13% lower under 2°C at the median.

## 2. Data

### (a) Observed climate and socio-economic data

We estimate economic impacts of climate variables on *per capita* economic growth using a panel of annual country-level observations. Data on economic growth is the change in the log of real GDP *per capita* (World Development Indicators, [22]) from 1960 to 2012 for 166 countries. Similar to BHM [11], we also consider the growth rate in agricultural value added (World Bank Development Indicators). Projections of future economic growth rates that are used to assess the climate impacts on levels of GDP *per capita* are obtained from the International Institute for Applied Systems Analysis (IIASA) SSP database [23] using the Organisation for Economic Co-operation and Development (OECD) scenarios of SSP1, 2, 4 and 5. In line with earlier econometric estimates of climate and growth *per capita* (e.g. BHM [11], DJO [12]), historical climate observations were obtained from Matsuura & Willmott [24] (here using v. 4) covering global land areas at a 0.5° resolution interpolated between stations. To map gridded climate observations to country levels, we weight climate data by gridded population data [25] at the same resolution. Population weighting, rather than area weighting, is more likely to accurately reflect the effects of climate on socio-economic activity [26].

Economic outcome data are generally measured at low frequencies (annually or quarterly), while many climate-related events that could impact economic growth occur over shorter time horizons (e.g. extreme precipitation events, storms or droughts). Thus, focusing on annual means of the metrics used—as has been the focus in the empirical impacts literature—risks missing most extreme weather events. We address this point by testing whether within-year monthly variation in temperatures and precipitation impact economic growth beyond nonlinear level effects of temperatures. In addition to annual means of temperature and precipitation, we therefore calculate the population-weighted variability (variance) of within-year monthly temperatures and precipitation, as well as the maximum and minimum monthly temperature and precipitation within each year for each country. We also assess the robustness of our results to within-year variability being computed at the grid-cell level, rather than after aggregation to country averages (see electronic supplementary material, section S6).

### (b) Climate projections under 1.5°C and 2°C warming: HAPPI projections

We derive projected monthly climate data from a large ensemble of atmosphere-only global circulation model simulations (HadAM3P) [27] that are driven by prescribed sea-surface temperatures (SSTs) and sea ice over one observed period and two distinct warming scenarios from the HAPPI set of projections. The historical period is based on observed SSTs and sea ice (OSTIA) [28] from 2006 to 2015 and corresponds to a current warming anomaly of about 0.9°C relative to pre-industrial levels. As a quasi-baseline scenario, this set-up is subsequently referred to as ‘no additional warming'. Both future warming periods use phase 5 of the Coupled Model Intercomparison Project (CMIP5) historical and projected SSTs and sea ice for a world 1.5°C and 2°C warmer than pre-industrial, where representative concentration pathways (RCPs) provide boundary conditions. The resulting CMIP5 ΔSSTs are added to the observed SSTs (see [15] for details of how HAPPI simulations are generated, including more details on the future sea ice generation). Radiative forcing is scaled from the RCP scenarios to match the specified warming targets. Anthropogenic aerosol emissions are set to one-third of current levels in 1.5°C and 2°C experiments. We caution that the associated aerosol effects may play a significant role with regard to simulated future rainfall changes. Climate projections were created using climate simulations aimed at arriving at specified warming anomalies rather than specified levels of radiative forcing. This approach was taken as the uncertainty in temperature response when using pre-specified radiative forcing (such as the RCP scenarios [29]) makes it impossible to differentiate between 1.5°C and 2°C of warming [30]. To illustrate, consider RCP 2.6, which has a median GMST increase of 1°C by 2100 when compared to 1986–2005 but has a likely range of 0.3°C and 1.7°C and hence covers both 1.5°C and 2°C warming.

**Figure 1.**
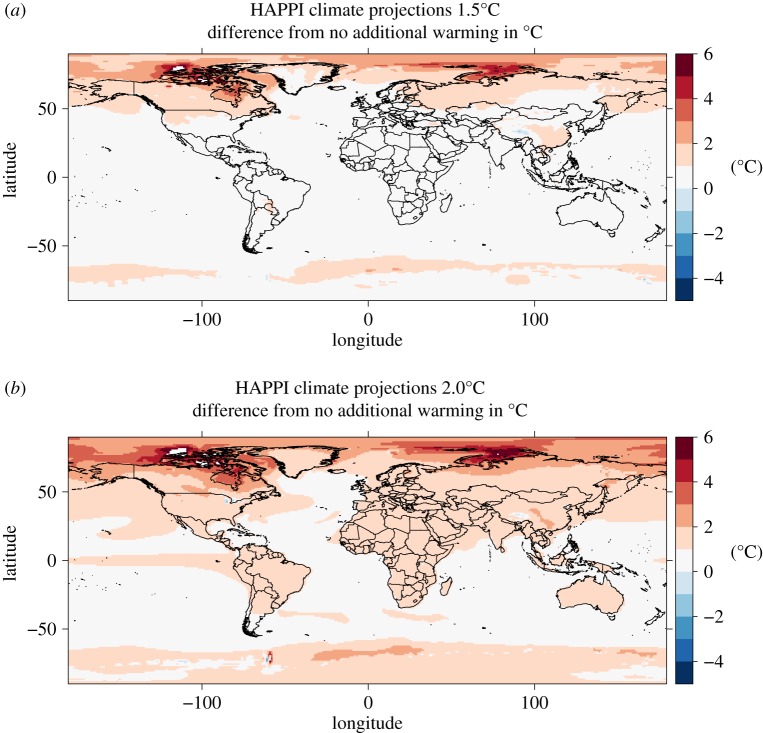
HAPPI temperature projections of 1.5°C (*a*) and 2°C (*b*) relative to no additional warming (having already observed an approximate 0.9°C increase in GMST relative to pre-industrial levels). (Online version in colour.)

**Figure 2.**
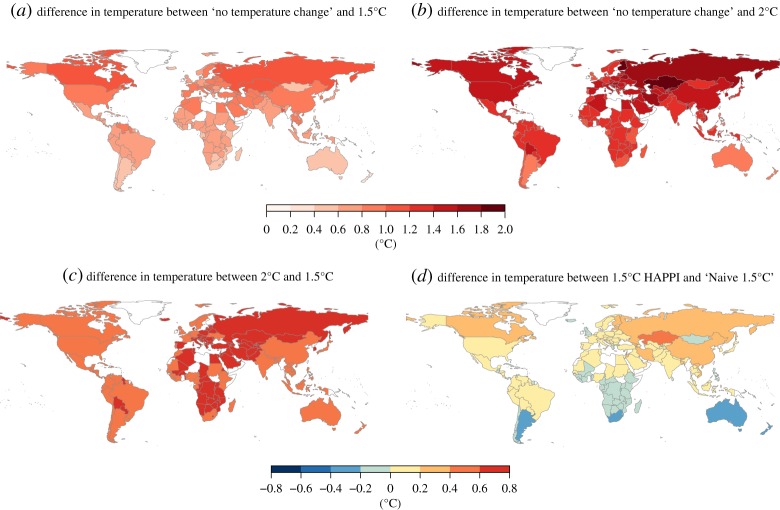
HAPPI projected changes in national and annual average temperatures, with national averages weighted by within-country population density for 1.5°C and 2°C relative to no additional warming (*a,b*). Differences between 1.5°C and 2°C are shown in (*c*), and difference between HAPPI 1.5°C and naive 1.5°C (by assuming all countries warm equally) is shown in (*d*). (Online version in colour.)

In order to provide a large number of ensembles of climate outcomes, HAPPI projections run with 105 ensemble members yearly for 10-year periods under both 1.5°C and 2°C (and 40 ensemble members for no additional warming) to incorporate natural variability such as the El Niño Southern Oscillation (ENSO) during this time (by construction, all scenarios represent natural SST and sea ice variability of the 2006–2015 historical period). We do not interpolate between the observed and projected periods and treat them independently. Figure 1 shows the HAPPI projected gridded temperatures under 1.5°C and 2°C warming relative to the baseline of no additional warming. We map gridded climate data for both the historical dataset and HAPPI projections to country levels by averaging over grid cells whose centroids lie within each country and weighting by population to match the socio-economic outcome data (figure 2). While some countries such as the United Kingdom or the Congo see a projected population-weighted warming close to the global average of 1.5°C, and some countries expect slightly below average warming (e.g. Australia and Argentina), most regions (particularly in the Northern Hemisphere) are expected to reach drastically higher temperatures (e.g. Greenland 2.2°C, Russia 2°C and Canada 2°C). This is because landmasses in general tend to warm much faster than oceans, which warm to about 1.02°C (relative to a 1.5°C global increase) on average according to CMIP5 estimates [15].

Naturally, aggregation onto a country level and annual frequency ignores much of the temporal and spatial information provided in HAPPI. Future efforts on estimating impacts could consider spatially disaggregated economic measures, as well as measures at higher temporal frequencies, such as industrial production and indices that explore effects of extreme weather events.

### (c) Comparing HAPPI 1.5°C to naive 1.5°C at a country level

We assess whether the HAPPI 1.5°C temperature projections are comparable to a naive 1.5°C warming scenario where average annual temperatures for each country are increased by adding 0.6°C (relative to 0.9°C of warming that has already occurred since pre-industrial levels for a total of 1.5°C). These naive 1.5°C temperature projections and HAPPI 1.5°C projections are resampled for each country and the differences compared (the resulting ranges for each country are shown in electronic supplementary material, figure S1).

While the range of temperature differences between actual 1.5°C and naive 1.5°C is wide (the 2.5% to 97.5% percentile range for most countries spans from −1°C to +1°C), the spatial pattern of differences highlights that HAPPI 1.5°C projects proportionally higher warming in the Northern Hemisphere relative to the naive 1.5°C, which assumes equal warming for every country (figure 2*d*), in line with established climate models [14]. Thus, while the uncertainties are large, a naive 1.5°C warming projection exhibits a systematic bias, under(over)-estimating country-level annual average temperature increases in the Northern (Southern) Hemisphere and overestimating ocean warming relative to warming over land.

## 3. Methods

### (a) Empirically estimated climate impacts on economic growth

Empirically estimating and subsequently projecting climate impacts faces distinct challenges: there are concerns about using historical data to estimate relationships for future projections as these relationships must be invariant to changes over time. To assess omitted shocks and stability over time, we allow for outlying country–year observations at every point in time using indicator saturation. Further, to construct socio-economic projections conditional on climate, we must assume that feedbacks from economic activity back onto climate are negligible. For the present study, we proceed under the assumption of weak exogeneity—studying climate impacts in isolation without explicitly modelling the underlying climate processes—and strong exogeneity—assuming an absence of feedbacks allowing the construction of conditional projections. However, given the strong evidence of economic activity affecting climate, the magnitude of feedbacks in empirical estimates and the potential failure of weak (and strong) exogeneity are open for further research [31].

Changes in climate may affect the growth rates or the levels of economic output or both. The existing evidence so far is not fully conclusive (see e.g. [8] and [32], for discussions), with most empirical macro-econometric studies focusing on and identifying growth-rate effects (see BHM [11] and DJO [12] for empirical tests to differentiate between growth and levels using distributed lag models).

The econometric literature that assesses the impacts of (climate and socio-economic) covariates on economic growth uses a quasi-experimental as well as a system-based econometric approach. In the quasi-experimental approach, the outcome variable (GDP growth *per capita*) is regressed on a set of treatment variables assumed to be exogenous, while most control variables are proxied through deterministic terms of country- and year-fixed effects together with deterministic trends. This yields estimates that aim to capture the overall effect of variables on the outcome of interest without explicitly modelling the channel of effects. Efforts have been made to identify the channels of impacts at industry or regional level—see Heal & Park [32] for an overview, and Hsiang *et al*. [33] (with discussion by [34]) for a detailed study of US impacts.

The system-based approach attempts to fully model the conditional expectation isolating the channel of impacts by including a large set of potential covariates (see e.g. the early cross-sectional studies by Barro [35] and Mankiw, Romer & Weil [36], with Durlauf *et al.* [37] providing an overview of growth econometrics). The vast set of potential determinants of growth has led to model selection approaches [38–40], and results highlight the importance of geographical factors beyond socio-economic covariates [41].

The literature on econometric models of climate impacts has predominantly focused on the quasi-experimental approach [42], and we follow this tradition here. While there are naturally concerns over omitted variables and structural change over time, here we extend the set of potential candidate climate variables relative to previous analyses and account for potential shifts and outliers by embedding the model in an indicator saturation framework. Further, for our analysis of relative impacts, we expect any omitted-variables problem to be less pronounced, as we assess projected impacts relative to each other under different scenarios [43].

#### (i) Estimation

Models are estimated as fixed-effects panel regressions including deterministic terms of country-fixed effects, year-fixed effects, and country-specific linear and squared time trends, similar to BHM [11] and DJO [12]. To account for potential outliers and unmodelled shifts in the data, we also estimate the models using impulse indicator saturation (IIS), applied in a dummy-variable fixed-effects panel. IIS adds a zero–one dummy variable for every observation (i.e. each country in each year) in the sample, where all but significant indicators are removed up until a target significance level *p_α_*, and remaining outlying observations are identified. IIS is closely related to robust estimation and goes beyond conventional fixed effects, as it fully saturates the regression model with a set of dummy variables, where each dummy uniquely identifies a single country--year observation (e.g. China in 1998). Thus, for the sample of 7007 observations (table 1), a total of 7007 dummy variables are added. This can be interpreted as a fixed effect for every single observation in the sample. As the model includes at least as many variables as observations, the model cannot be estimated directly; instead, estimation is conducted through block partitioning of indicator variables. If there are truly no outlying observations, then, despite the inclusion of a large candidate set of dummy variables, the distribution of variables not selected over remains unaffected beyond small efficiency effects (the asymptotic theory of block partitioning and IIS is discussed in [17,19,44]). IIS can act as a flexible test for model misspecification without requiring prior knowledge of the form of misspecification, or which observations deviate from the estimated model. If few to no indicators are retained (close to expected value of *p_α_* × *S*, where *S* denotes the total number of dummy variables included), then we cannot reject that the model is well specified. However, if more indicators are retained than expected, this can be informative about the underlying unmodelled characteristics of the data (see [45] for a formal discussion of IIS misspecification testing). IIS has been applied to various settings, including the identification of unknown volcanic eruptions in historic temperature records [46,47], to evaluate climate models [48], assess economic forecasts [49] and test the robustness of panel models of unemployment [50]. For the present analysis, IIS is implemented using the R-package ‘gets' [20]. All models are estimated as a dynamic panel^1^ with the general model specified as
3.1
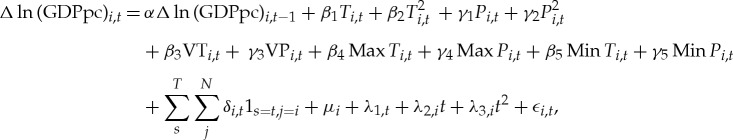

where 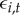 is an iid (independent and identically distributed) mean-zero error term, VT*_i_*_,*t*_ denotes the variance of monthly temperatures for country *i* in year *t*, and Max *T_i_*_,*t*_ (Min *T_i_*_,*t*_) refers to the maximum (minimum) monthly average temperature for country *i* in year *t*. Precipitation variables (*P* and VP) are constructed in an identical manner. Coefficients *δ_i_*_,*t*_ capture potentially outlying country–year observations through IIS, where the saturated set of indicator variables is reduced using model selection at a specified target significance level *p_α_*, with an expected false retention rate (gauge) for IIS given by *p_α_* × *S*. We estimate multiple versions of (3.1) specified as: model M1 (including within-year variability without IIS), M2 (including within-year variability with IIS), M3 (excluding within-year variability with IIS), M4 (modelling growth in agricultural value added without IIS) and M5 (modelling growth in agricultural value added with IIS).

**Table 1.** Estimation results from equation (3.1) using within-year variables (Max, Min, Var) together with impulse indicator saturation (IIS) for growth in GDP *per capita* (models M1, M2, M3) and growth in agricultural value added (models M4 and M5). Standard errors are given in parentheses. Significance: *5%, **1% and ***0.1%. IIS applied at significance level of *p_α_* = 0.001, with an expected number of outliers (under the null) of 0.001 × 7007 ≈ 7. Models include country-, year-fixed effects, country-specific linear and squared time trends.

dep. variables	*y_i_*_,*t*_ = Δln(GDPpc)*_i_*_,*t*_			*y_i_*_,*t*_ = Δ%GDPAgric*_i_*_,*t*_	
indep. variables	M1: within yr	M2: within yr + IIS	M3: no within yr + IIS	M4: within yr	M5: within yr + IIS
*y_i_*_,*t*−1_	0.1243	0.1604	0.1598	−0.2706	−0.237
	(0.0121)***	(0.0099)***	(0.0099)***	(0.0142)***	(0.0122)***
Temp.	0.0163	0.0115	0.0083	−0.0013	0.0074
	(0.0039)***	(0.0029)***	(0.0023)***	(0.0083)	(0.007)
Temp. squared	−0.0005	−0.0004	−0.0004	−0.0004	−0.0005
	(0.0001)***	(0.0001)***	(0.0001)***	(0.0002)	(0.0002)**
Precip.	0.0014	0.0015	0.0011	0.0102	0.0082
	(0.0013)	(0.0009)	(0.0009)	(0.0025)***	(0.0021)***
Precip. squared	0.0001	0.0001	0.0001	−0.0003	−0.0002
	(0.0001)	(0.00005)*	(0.0001)	(0.0001)***	(0.0001)***
Var(Temp.)	0.0002	0.0002		−0.0022	−0.0018
	(0.0002)	(0.0001)		(0.0004)***	(0.0003)***
Max(Temp.)	−0.0007	−0.0001		0.0021	0.0006
	(0.0013)	(0.001)		(0.0025)	(0.0021)
Min(Temp.)	−0.0008	−0.00005		−0.0037	−0.003
	(0.0009)	(0.0007)		(0.0018)*	(0.0015)*
Var(Precip.)	−0.0001	0.0001		0.0001	0.0001
	(0.0001 )	(0.0001 )		(0.0001)	(0.0001)
Max(Precip.)	0.0003	0.0001		−0.0002	−0.0005
	(0.0003)	(0.0002)		(0.0005)	(0.0004)
Min(Precip.)	0.000006	0.0003		−0.0002	0
	(0.0007)	(0.00005)		(0.0013)	(0.0011)
observations	7007	7007	7007	5034	5034
log likelihood	10820.52	13106.66	13101.12	5398.51	6396.7
no. outliers (IIS)		81	81		73

### (b) Projecting impacts of climate scenarios using empirical impact estimates

Using the econometric estimates of climate impacts from (3.1), we project the expected economic response conditional on HAPPI climate scenarios assuming that the estimated historical relationships are stable and unchanged during the projection horizon (implicitly not accounting for unpredictable technological progress). On the climate side, this assumption is supported by evidence that significant nonlinear changes to the climate system, i.e. tipping points which would fundamentally change the underlying dynamic of the relationship between climate and the economy, are unlikely to occur below 2°C [52]. The coefficients on temperature variables are scaled by the autoregressive coefficient and thus represent the equilibrium impact.^2^ We first generate projections of climate impact on growth rates without assuming a specific decade in which the target temperatures are reached. Subsequently we construct projections of the level of GDP *per capita* under the assumption that the target temperatures are reached at the end of the century.

#### (i) Quantifying uncertainties

Quantifiable uncertainty about future projections within the model stems from uncertainty on the econometric parameter estimates of the growth response to climate changes (the estimated coefficients in equation (3.1)), and uncertainty about future climate itself. The range of uncertainty presented here, however, is probably a lower bound, as there is high non-quantifiable uncertainty on whether the underlying model is correct. To quantify econometric estimation uncertainty (assuming a correct model), we resample estimated coefficients by creating draws from a multivariate normal distribution of the coefficients of interest (the autoregressive coefficient and relevant climate coefficients) using the estimated covariance matrix in the regression model.^3^ To quantify uncertainty around climate outcomes, we use the sampling uncertainty over land areas, established by virtue of individual ensemble members under the same SST and forcing conditions (1.5°C, 2°C and no additional warming) from HAPPI; an uncertainty unaccounted for by existing econometric climate impact projections.^4^ To assess the relative contributions of sources of uncertainty, we repeat projections of growth rates by sampling (with replacement) over the estimated impact coefficients, climate outcomes or both. In other words, we assess the relative contribution to overall uncertainty by allowing for uncertainty over the economic impact function, climate outcomes or both (see also [54] for a discussion of economic and climate uncertainty).

#### (ii) Projecting growth-rate impacts

Owing to the nonlinear functional form of temperature impacts on economic growth in (3.1), the temperature level of each country affects its projected growth. Simulated climate values from HAPPI are, therefore, matched to the estimation data by correcting for a climate model bias in mean temperatures on a country-by-country level. For each country, we compute the average observed temperature over 2006–2014 as well as the average simulated temperature of the respective country in the HAPPI ‘no additional warming’ simulation designed to match the same time span.^5^ The difference is subtracted from the HAPPI simulations to correct for model bias. To project the change in economic growth under different temperature scenarios, we resample over our econometric coefficients, base temperature outcomes and projected temperature outcomes. The base temperature outcomes are taken from the HAPPI simulations under no additional warming.^6^ Projected temperature impacts on economic growth are assessed relative to the baseline ‘no warming’ scenario and are independent of any time period (and SSP scenario). Relative growth impacts are solely a function of the levels and differences of climate variables (temperature), and thus independent of the year when the target temperature is reached.

#### (iii) Projecting level impacts

We project the impact of temperature changes on the level of GDP *per capita* by assuming that the target temperature (1.5°C or 2°C) is reached in the year 2100. Temperature impacts are assessed relative to baseline economic scenarios SSP1, 2, 4 and 5. Because no single SSP scenario was tailored to 1.5°C warming, we report SSP2 in the main text and provide SSP1, 4 and 5 in the electronic supplementary material. SSP3 is excluded as it is inconsistent with limiting warming to 1.5°C [21]. To project the level of *per capita* GDP, countries are initialized with their observed level of *per capita* GDP in the year 2010 (the year which overlaps both SSP projections and the estimation sample), and subsequently GDP is projected until 2100 using the SSP GDP and population growth rates. Temperatures are assumed to increase linearly until the target temperature is reached; we also consider a nonlinear increase in the electronic supplementary material, section S4.^7^ The impact of each HAPPI scenario is assessed against the SSP scenario by subtracting the projected climate growth-rate effect from the SSP scenario growth rate for each country. Uncertainties around the projected growth rate are derived from resampling over both model coefficients and climate projections. This results in a large sample of potential GDP outcomes for each country. Global average GDP *per capita* is constructed using a population-weighted average in each projection year.

## 4. Results and discussion

### (a) Impact estimates using indices of within-year climate variation and outlier detection

**Figure 3.**
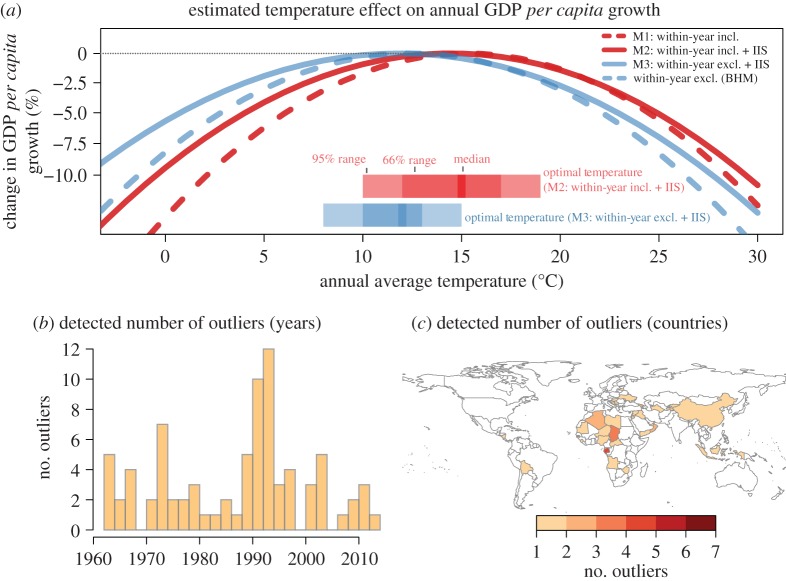
Estimation results (based on table 1). (*a*) Estimated quadratic impact of annual-mean temperatures on economic growth with (red, dashed) and without (blue, dashed) variables for within-year temperature and precipitation measures, as well as using impulse indicator saturation to detect and control for outlying country–year observations (solid). Detected outliers through IIS (in model M2) are shown over time (*b*) and over countries (*c*). Model results excluding within-year variables and without IIS are shown as reference corresponding to an updated estimate of BHM [11] (blue dashed). (Online version in colour.)

There is little evidence of within-year climate variation showing a significant impact on economic growth beyond an overall nonlinear effect of temperatures (table 1 and figure 3). Temperature and precipitation variability as well as maximum and minimum values are not significant in the panel regressions (see electronic supplementary material, section S6 for results using variability calculated at the grid-cell level). Detecting outliers and breaks through IIS results in a large set of outlying country--year observations being identified. The number of retained impulses is significantly higher than expected by chance (81 retained versus 7 expected at *p* < 0.001 using the normal test in [45]) and identifies periods and regions falling outside the model specification. These are concentrated around central Africa and eastern Asia, with a peak in the early 1990s (figure 3*b,c*). These could be indicative of measurement errors as well as unmodelled impacts of the collapse of the Soviet Union.

Controlling for these outlying observations reduces the slope of the estimated temperature curve below the optimal temperature (suggesting reduced impact of temperatures on countries with low average annual temperatures). Including controls for within-year variability, while not individually significant, shifts the peak of the curve (and thus optimal temperature) to the right, slightly dampening the impact on economic growth for countries with higher average annual temperature. However, the detected outliers and within-year climate variables have little effect on the overall inverted U-shaped nonlinear impact of temperatures on GDP growth. We do, nevertheless, find that growth in agricultural production is significantly affected by temperature and precipitation variability, where the nonlinear level effect becomes insignificant once within-year variability is included. Variability in precipitation has detrimental effects on agricultural GDP growth, potentially through reduced predictability or extreme events such as droughts and floods [55].

### (b) Projections of impacts

Given the statistical insignificance of within-year temperature and precipitation variability impacts on growth in GDP *per capita*, we proceed to project impacts using the estimated nonlinear (quadratic) impact of temperature levels from model M2 (including within-year variables as controls and IIS) to construct projections under 1.5°C and 2°C warming. We refer to growth-rate projections as ‘statistically significant' when the 95% (2.5%–97.5%) percentile range of projections falls outside of zero.

#### (i) Growth projections

**Figure 4.**
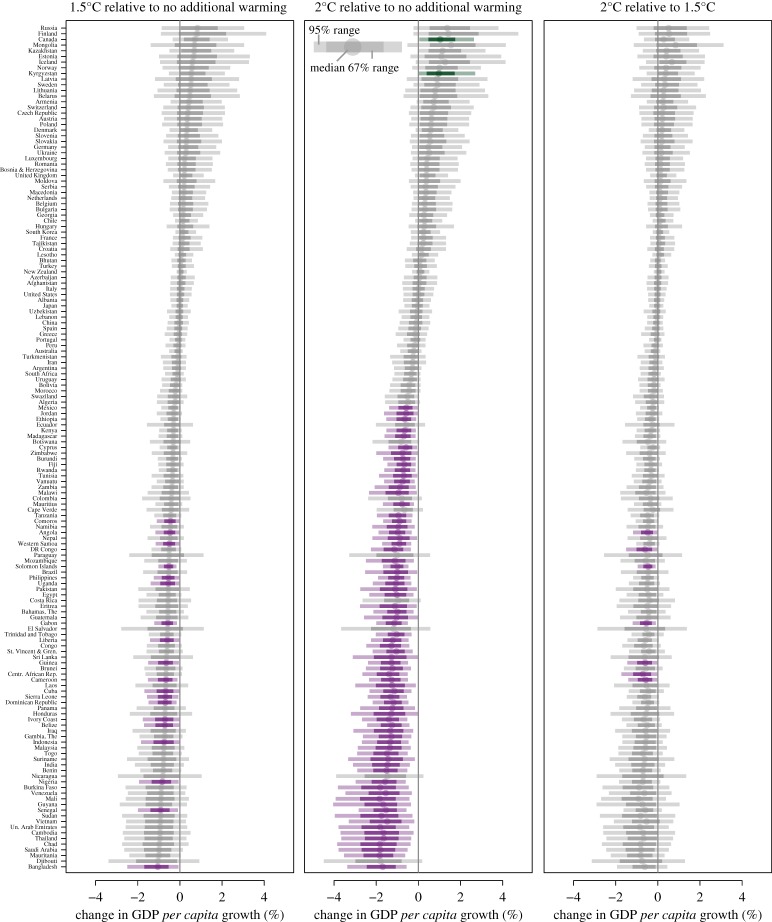
Growth-rate projections combining econometric estimates of temperature impacts on economic growth with HAPPI projections of 1.5°C and 2°C warming relative to pre-industrial levels. (*a*) 1.5°C relative to no additional warming, (*b*) 2°C relative to no additional warming and (*c*) 1.5°C relative to 2°C. Plot shows the median (dots) projected relative impact, together with the 2.5%–97.5% percentile range (light shaded) together with the 17%–83% range (67%, darker shaded) accounting for both climate and econometric estimation uncertainty. Countries are ordered by the median projected impact of 1.5°C. Countries where the 95% percentile range falls below (above) zero are shown in purple (green).

**Figure 5.**
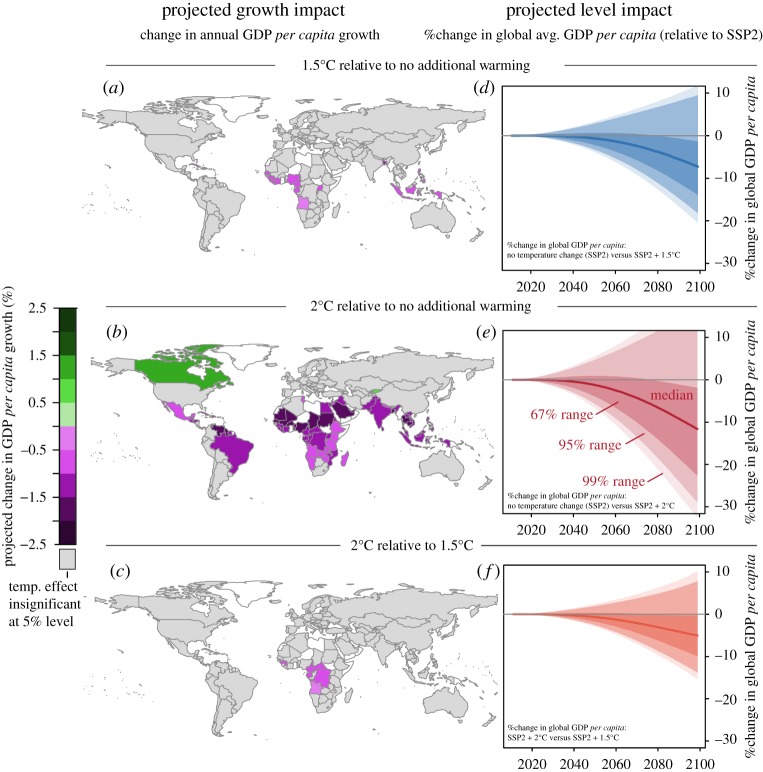
Growth-rate (left) and level (right) projections combining econometric estimates of temperature impacts on economic growth with HAPPI projections of 1.5°C relative to no additional warming (*a,d*), 2°C (*b,e*) and 1.5°C compared to 2°C warming (*c,f*). (*a–c*) Maps of projected median impacts on economic growth Δln(GDP *per capita*), where countries are coloured if their 95% range falls outside of zero (as determined from figure 4). Countries where the 95% percentile range falls below (above) zero are shown in purple (green). (*d–f*) The projected percentage difference in global average GDP *per capita* relative to the baseline SSP2 scenario under 1.5°C (*d*) and 2°C (*e*) warming, as well as 1.5 relative to 2°C (*f*). Target temperatures are assumed to be reached in 2100 for the level projections. Shading corresponds to the 67% range (darkest), to 95% and 99% (lightest), with the median projection given as the solid line.

Projections of economic growth under 1.5°C and 2°C are shown in figures 4 and 5*a*–*c* accounting for both econometric estimation and climate uncertainty. Most countries exhibit a projected decline in economic growth under 1.5°C relative to no additional warming (baseline growth); however, this decline is not statistically distinguishable for the majority of countries, as the 2.5%–97.5% range spans zero. In turn, projected impacts from 2°C warming are statistically lower relative to no additional warming for a large set of countries (figures 4 and 5*b*), with median projected declines in annual economic growth of up to −2%. No country shows a significantly positive growth effect under 1.5°C, and the effects under 2°C are only positively statistically significant for two countries (Canada and Kyrgyzstan). (The electronic supplementary material, section S5 shows additional maps of the significance of projected impacts when considering both a 95% as well as a 67% range of significance.)

It is important to emphasize that the (squared) functional form of the estimated impact function implies that, whether the point estimate of a growth impact is positive or negative depends on the annual average baseline temperature of a country and the estimated optimal temperature. Projections have to be considered with their corresponding uncertainties, as a slight bias in temperatures (or shape of the estimated impact function) that moves a country away from the optimal temperature will influence whether the projected point effect is negative or positive. Figure 3 shows the distribution of the optimal temperature for two models (M3 and M2) with their 95% range spanning 7°C to 15°C (and 10°C to 18°C), respectively.

The geographical impact of 1.5°C and 2°C is non-uniform. While the warming pattern of 1.5°C suggests a disproportionate increase in temperatures in the Northern Hemisphere (figures 1 and 2), the impact on economic growth is projected to predominantly affect countries around the Equator and the Southern Hemisphere (figure 5*a*–*c*). Previous studies (e.g. early work by [41]) also found that countries in the tropics are probably more vulnerable to climate variations based on historical relationships.

**Figure 6.**
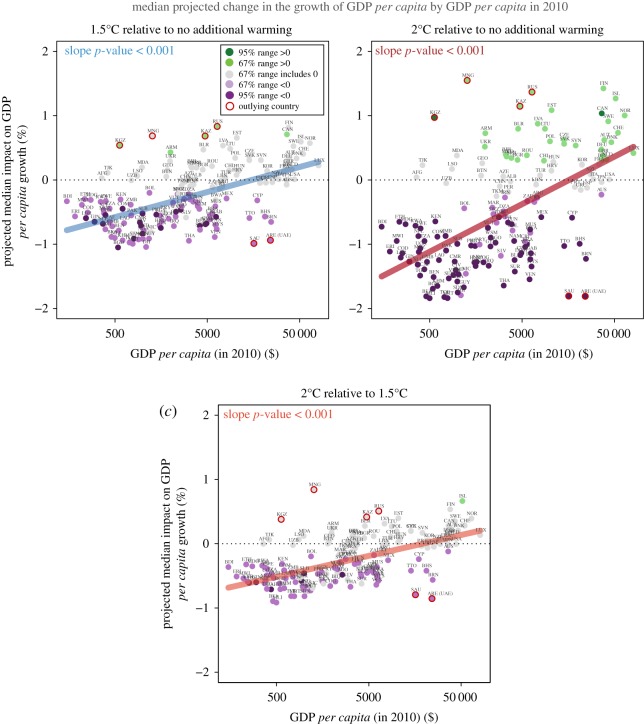
Relationship between projected temperature impacts on GDP *per capita* growth and present-day income levels as given by GDP *per capita* in 2010. (*a*) Median projected impacts under 1.5°C relative to no additional warming, (*b*) 2°C relative to no additional warming and (*c*) 1.5°C relative to 2°C. Slopes are given by robust ordinary least-squares estimates with outlying countries (dark red) identified and removed using IIS (at *p_α_* = 0.05). Countries with lower GDP *per capita* exhibit statistically greater negative impacts, and this relationship becomes more severe for higher warming scenarios, suggesting higher projected inequality across countries.

Projected impacts on economic growth are significantly correlated with present-day GDP *per capita*, as countries with lower current *per capita* incomes are projected to experience stronger negative growth effects than countries with higher incomes (figure 6). The relationships between projected impacts and current income levels are highly significant for all three comparisons of warming scenarios (between 1.5°C, 2°C and no additional warming), and the estimated slope between impacts and income becomes steeper for larger temperature changes—the warming effects diverge more across incomes as temperatures increase, suggesting an increase in inequality across countries. These differing impacts on economic growth rates reduce the likelihood of future economic convergence across countries [56].

Even though the impacts of 1.5°C on growth are statistically similar to no additional warming, and the impacts of 2°C are statistically different from no change, the growth impacts of the two temperature scenarios (1.5°C and 2°C) themselves are not statistically distinguishable (figure 4*c*). This can be reconciled by noting that the 95% range of 2°C falls outside of zero but spans the median impacts of 1.5°C. The median difference in projected economic growth is, nevertheless, up to 1% higher under 1.5°C relative to 2°C for the majority of countries.

#### (ii) Level projections

Projections of the difference in level of GDP *per capita* under 1.5°C and 2°C warming are shown in figure 5*d*–*f* for SSP2 (SSP1, 4 and 5 are reported in the electronic supplementary material, section S3). The uncertainties in growth rates compound when assessing projections of the levels of GDP *per capita*, as the global 95% range spans zero in all three warming scenario comparisons. However, median projected global GDP *per capita* is lower for both 1.5°C (median reduction of approximately 8% in 2100) as well as 2°C (median reduction of approximately 13% in 2100) relative to no additional warming. Comparing 1.5°C to 2°C, median global average GDP *per capita* is projected to be approximately 5% lower under 2°C in 2100.

### (c) Quantifiable sources of uncertainty

**Figure 7.**
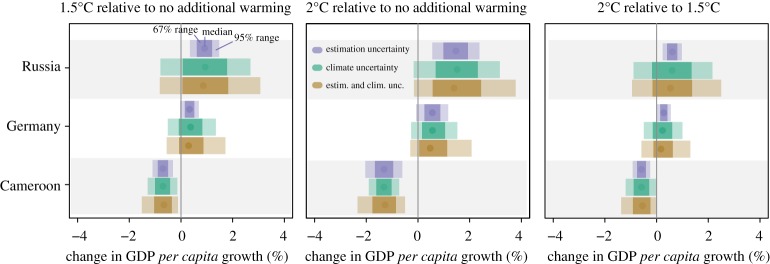
Quantifiable uncertainty around projected growth-rate impacts. Showing the 95% range (light shaded), 67% range (darker) and median (points) projected growth-rate impact when only considering econometric uncertainty (purple), climate uncertainty (green) and both (brown).

The above results show the projected impact of taking both econometric estimation uncertainty as well as climate uncertainty into account. To assess the contribution of individual uncertainties, figure 7 shows the range of uncertainties implied by different sampling schemes for three representative countries. First, climate is held fixed at the ensemble means for each country and the only source of uncertainty is assumed to be econometric estimation uncertainty; second, we assume the impact function is known (fixed at the maximum-likelihood estimate) and climate is uncertain; and third, we consider both uncertainties jointly (as in figures 4 and 5). As the results in figure 7 show, ignoring any one source of uncertainty leads to spurious precision on top of unquantified model uncertainty. Growth impacts may be deemed significant if only econometric estimation uncertainty is taken into account (e.g. for Russia in figure 7); however, they are insignificant once climate uncertainty is considered. No single source of uncertainty appears to dominate another, further highlighting the importance of accounting for both climate and econometric estimation uncertainty. Naturally the uncertainty for individual countries can be high, as model results are aggregated relying on annual measures at a country level, masking any heterogeneity in responses within countries. For example, in the country-level panel analysis here, the uncertainty around projected growth impacts on the USA is high, while recent within-country estimates of US climate impacts [33] project a significant negative impact of roughly 1.2% on US GDP for each degree of warming along with higher within-country inequality. This magnitude of effects is comparable to results presented in figure 5, suggesting median reductions in global average *per capita* GDP of 13% for a 1.1°C (from 0.9°C to 2°C) increase in GMST, noting that this number is derived from global estimates and includes countries with higher projected impacts.

A further source of uncertainty beyond the estimated model is that our projections do not formally include impacts from sea-level rise (historical damages are difficult estimate from this metric—sea-level rise will enhance the impact of rare-event-driven storm surges and periodic tidal flooding)^8^ and other unanticipated climate events or tipping points/elements (e.g. [58]). For a business-as-usual world, the 2°C threshold is likely to be reached around 2040. In this case, global sea-level is projected to be 20 cm higher (relative to 1986–2005 [59]) but the response will be nonlinear in time and non-uniform in space (e.g. [60,61]).

## 5. Conclusion

Using a set of dedicated climate simulations to project GMST increases of 1.5°C and 2°C, we find greater warming in population-weighted temperatures in the Northern Hemisphere than expected from a uniform increase in average temperatures, in line with established climate models. Empirically estimating the impacts of climate variables on GDP *per capita* growth, we find that a nonlinear relationship of temperature and GDP growth is robust when accounting for within-year monthly temperature and precipitation variability, as well as monthly maximum and minimum values of temperatures and precipitation and outliers. Temperature changes have little impact on growth for countries falling around an optimal annual average temperature, while there appear significant temperature effects for countries exhibiting very high (and low) annual average temperatures consistent with BHM [11] and DJO [12]. Our results, however, show that positive growth effects for colder countries are dampened when controlling for outliers, and within-year variability plays a significant role in growth of agricultural production. This agricultural effect is probably not evident in the overall growth measure, as agricultural activity as a share of overall GDP has been declining for most countries in the sample [22].

The projected impacts on economic growth of 1.5°C warming relative to no additional warming (and relative to 2°C) are uncertain, as the range of likely outcomes within the model is large when accounting for both climate and econometric estimation uncertainty. Our findings suggest that the impact of 1.5°C is close to indistinguishable from current conditions, while 2°C warming implies significantly lower projected economic growth for a large set of countries.

While statistically we cannot rule out that the projected impacts of 1.5°C relative to 2°C are similar (as their confidence ranges overlap), the median projected growth in GDP *per capita* (and subsequently projected level of GDP *per capita*) is lower under 2°C than under 1.5°C warming for the majority of countries. Projected losses are significantly higher for low-income countries across both temperature scenarios, suggesting higher economic inequality across countries as a result of projected future climate change. In the case of projected levels of GDP *per capita* by the end of the century, average GDP *per capita* is 8% and 13% lower at the median for 1.5°C and 2°C, respectively, relative to base scenarios. We emphasize that these results are solely the projected net effect of temperatures assuming the estimated relationships are stable. Beyond the quantifiable uncertainty within our model, the overall uncertainty of economic growth under hypothetical temperature scenarios is large, yet results suggest that significant benefits are likely to arise from lower levels of warming, providing further support to pursue efforts using stringent emission adjustments [62] to limit GMST warming to 1.5°C rather than 2°C or higher.

## Supplementary Material

Supplementary Material - Details on Methods and Data

## Footnotes

1 Given the long time frame of estimation (*T* ≈ 50 years), the bias in dynamic panel estimation will be small [51].

2 Equilibrium impacts are thus given by the long-run solution [53] of the model. The estimated autoregressive coefficient , while highly significant, is estimated to be around 0.17 across models, suggesting little persistence in the dependent variable.

3 An alternative is to resample over estimation data or residuals; however, due to the computational requirements of indicator saturation, we restrict our analysis to resampling from the multivariate distributions of estimated coefficients under the assumption of normality, which is appropriate having removed identified outliers through IIS, and thus moved the residuals closer to a normal distribution. Coefficients are resampled 2000 times in the projections reported here.

4 Climate outcomes are resampled 2000 times with replacement out of the available individual ensemble outcomes.

5 Some countries do not have full observations over 2006–2014; in that case the mean is constructed over the years that are available over this time span.

6 Using observed actual temperatures rather than HAPPI actual temperatures confounds any implicit difference between observations and future projected climate outcomes.

7 A linear increase in temperatures appears roughly consistent with IPCC projections ([14], see figs 11.25 and 12.1); however, we consider a logarithmic increase in the electronic supplementary material.

8 Observed global average sea-level rise over the estimation period 1960–2012 is around 0.15 m [57].

## Data accessibility

Data are available from the referenced sources or from the authors on request. Electronic supplementary material is available online at rs.figshare.com.
