# Supplementary material for: Uncertain impacts on economic growth when stabilizing global temperatures at 1.5°C or 2°C warming
Source: Philos Trans A Math Phys Eng Sci. 2018 Apr 2;376(2119):20160460. doi: 10.1098/rsta.2016.0460 (PMC5897830; doi:10.1098/rsta.2016.0460)
Supplement: Supplementary Material - Details on Methods and Data [file rsta20160460supp1.pdf]

PHILOSOPHICAL
TRANSACTIONS A

Phil. Trans. R. Soc. A.
DOI 10.1098/rsta.2016.0460

Electronic Supplementary Material

Uncertain impacts on economic growth when stabilizing global temperatures at 1.5°C or 2°C warming

Felix Pretis, Moritz Schwarz, Kevin Tang, Karsten Haustein, Myles R. Allen

Article doi: 10.1098/rsta.2016.0460

Index

S1: Comparison of HAPPI 1.5°C to Naïve 1.5°C

S2: Differences to BHM (Burke, Hsiang, and Miguel, 2015)

S3: Alternative SSP Scenarios

S4: Nonlinear Temperature Increase

S5: Mapping Uncertainties in Impacts

S6: Alternative Measure of Within-Year Variability

Supplementary Section S1: Comparison of HAPPI 1.5 to Naïve 1.5°C

Figure S1 shows the 95% range, the 66% range and median difference in projected temperature change by country between HAPPI 1.5°C and a naïve 1.5°C constructed by adding 0.6°C to the average annual country temperature under the modelled current conditions. While the ranges are large, HAPPI 1.5 generally leads to higher warming than the naïve estimate and particularly the spatial distribution of warming differs between the HAPPI 1.5 and naïve projections (see Figures 1 and 2).

Figure S1: Comparing HAPPI 1.5C to 'Naïve 1.5C'

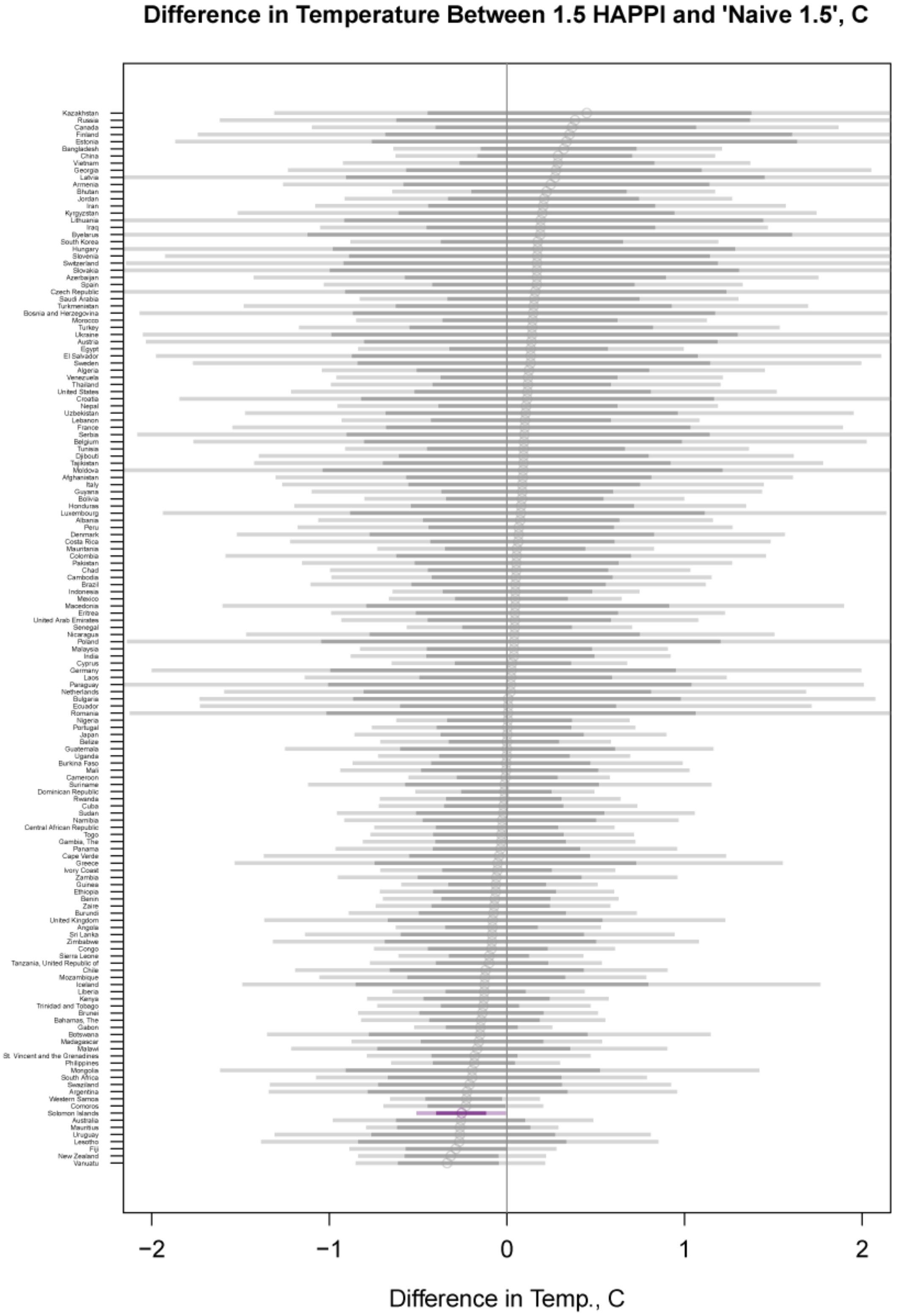

Supplementary Section S2: Details on Methodology and Differences to BHM

We build on the empirical literature estimating the impacts of climate variables onto economic growth where our model is closely related to the approach of BHM (Burke, Hsiang, and Miguel, 2015) with notable differences discussed in more detail here.

S2.1) Estimated Empirical Model and Observed Climate Data

BHM estimate the response of per-capita GDP growth using a global panel dataset including the level of temperature and precipitation as their main climate controls. Here we update and expand their analysis in a number of ways. Climate data used for estimation here is version 4 of the Matsuura and Willmott dataset relative to version 3 in BHM. Comparing the two versions reveals significant divergences after population-weighting, as shown in Figure S2. The method of aggregation and the population data are consistent with BHM methods and country level and data from BHM can be recreated using version 3. Figure S2 shows the differences between version 3 and 4 of the historical climate observations by magnitude (top) and by country over time (bottom). For greater clarity, outliers are highlighted.

Figure S2: Differences between Population-Weighted Temperature Series – Version 4 relative to Version 3

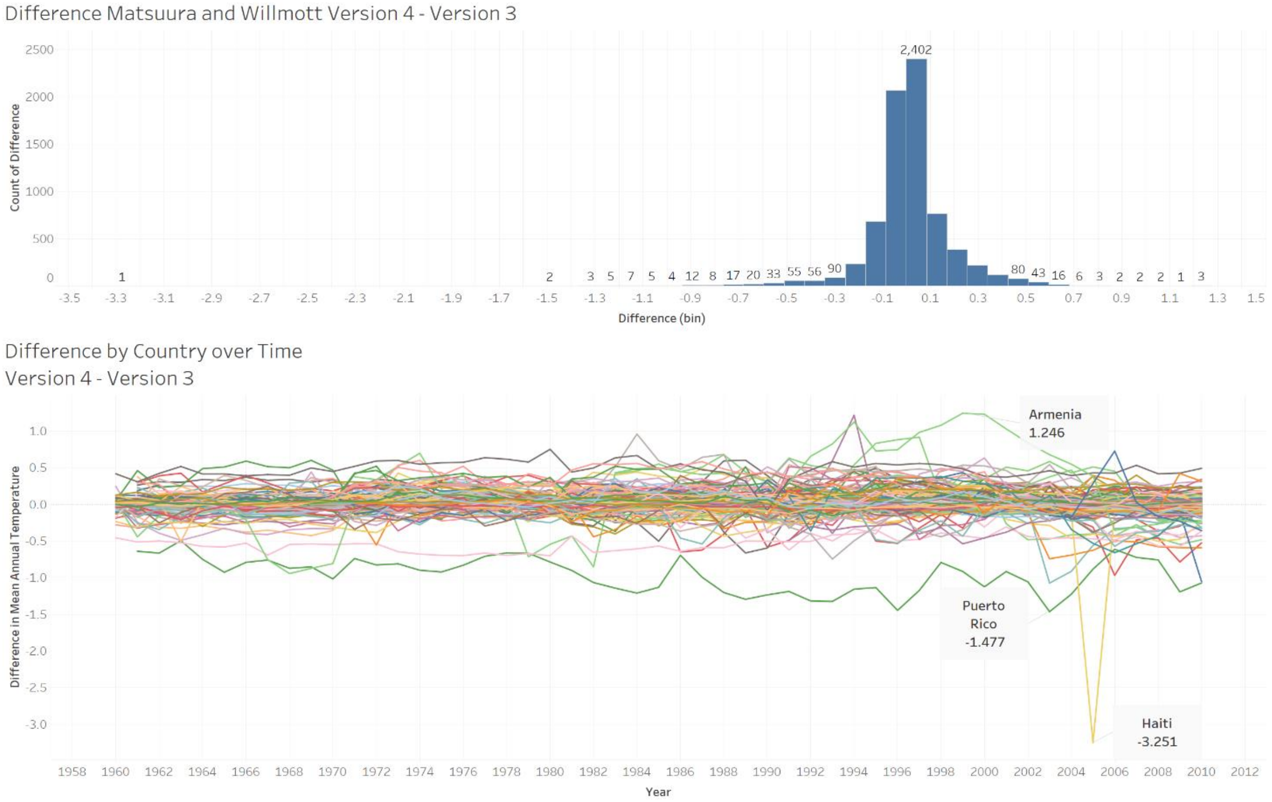

Alternative climate variables were calculated to test the effects of extremes and within-year variability. Since historical observations used in this paper are only featured in monthly data, widely used extreme weather indices could not be calculated (such as those suggested by Karl *et al.*, 1999). Instead we use within-year maximum and minimum precipitation and temperature, as well as the variance of monthly temperature and precipitation within each year. Using monthly observations of temperature and precipitation, grid cells were aggregated to a country level by averaging over grid cells whose centroids lie within each country for each month and subsequently the month with the highest (lowest) temperature was selected for this country's max (min) value that is used in the model. Temperature and Precipitation variance was calculated by calculating the variance of country-aggregated monthly values, while annual means were calculated by taking the mean of all available months. Despite a relatively high spatial resolution of 0.5°, a number of small (mostly island) countries and territories had to be excluded from the study, due to the lack of observational data in the dataset. Countries without complete observations across variables are removed, resulting in a final sample of 149 countries.

The resulting panel model is then saturated with a full set of indicator functions, and model selection applied to remove all but significant indicators at a target significance level p=0.001 using the R-package gets (Pretis, Reade, and Sucarrat, 2017).

S2.2) Projections

BHM consider a projection of their estimated climate impacts onto the level of GDP per capita using the business-as-usual RCP 8.5 scenario. BHM do not account for climate uncertainty in their projections as they use the ensemble mean climate for temperature projections. Individual countries appear to exhibit drastic GDP effects (Figure 4 in BHM) when the point-estimate is used for projection despite the estimated impact likely not being statistically different from zero. Here we consider projections tailored to 1.5°C and 2°C warming, together with econometric and climate uncertainties.

Climate projections under 1.5° and 2°C warming are taken from the ‘Half a degree Additional Warming, Prognosis and Projected Impacts’ (HAPPI) project (see Mitchell *et al*, 2017 for detailed set-up and also http://www.happimip.org/ for further information). In order to match the spatial dimension and resolution of the climate data to the population data, we resample the output of the atmosphere-only HadAM3P model from a 1.875°x1.25° resolution to a 1°x1° resolution using bi-linear weights. Country values and extreme value indicators were calculated with the same methodology as the historical data.

Since the sensitivity of the initial temperature effects on GDP per capita is relatively high, HAPPI projections were bias-corrected to match historical observations and to avoid significant jumps in the transition from observations to projections. Bias correction was undertaken over the period of 2006-2014 where both HAPPI projections and observational data was available by adjusting for the mean difference in values from the HAPPI projections over the entire period.

Level projections using SSP growth rates are initialised with observed historical GDP per capita values in 2010 (the year when projections and observations overlap). Subsequently GDP per capita is projected to 2100 using SSP scenario population and GDP growth rates.

Supplementary Section S3: Shared Socio-Economic Pathways (SSPs)

To project impacts of 1.5C and 2C on level of GDP per capita to the year 2100, we rely on growth projections from the SSP scenario family. SSP2 is reported in the main text, projections under alternative scenarios (SSP1, 4, and 5) are shown here in Figure S3. Scenario data is available from the SSP database (IIASA, 2016). Population growth factors include age and sex specific proportions of educational attainment, and projects population growth on the basis of fertility rates, mortality rates, and migration. For GDP projections, we use the OECD modelling framework based on a two-sector economy using an augmented Solow growth model.

Figure S3: Projected Impacts under SSP1 (top row), SSP4 (middle row), and SSP5(bottom row)

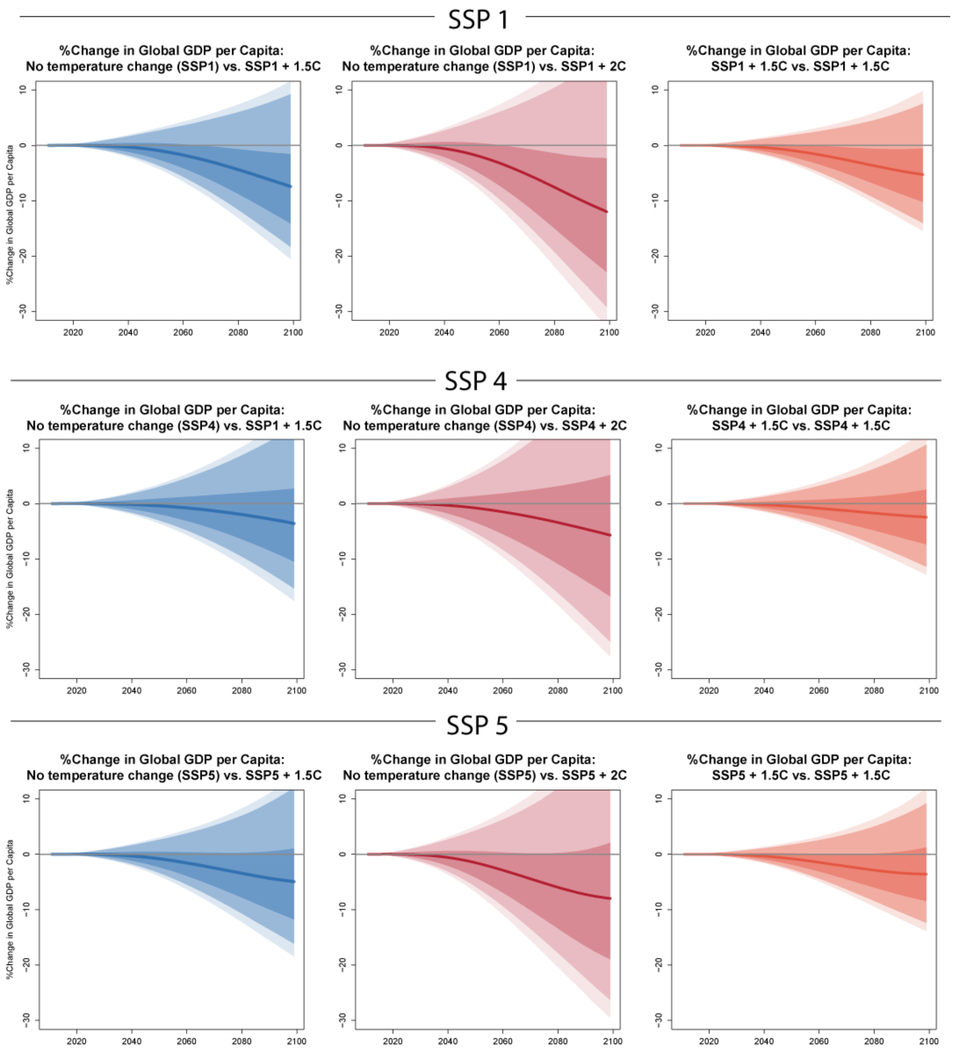

Supplementary Section S4: Non-linear temperature increase in projections

To assess the impact of assuming non-linear temperature increase over the projection period, we compare linear results to a logarithmic increase in temperatures until the target temperatures for each country are met (see Figure S4.1).

Figure S4.1: Linear and logarithmic temperature path over the projection period for a hypothetical increase of 1.2C.

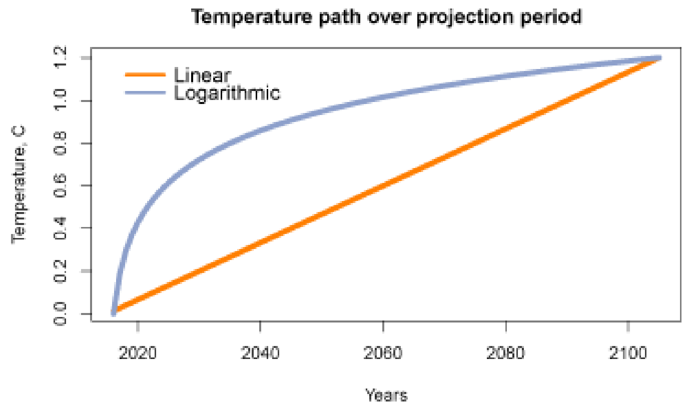

The projected impact onto levels of GDP per capita is shown in Figure S4.2. Projected impacts are larger under logarithmic increase as the temperature increase occurs more rapidly at the beginning, and the amplified growth rate effect at the start of the projection period compounds in levels for the remainder of the projection period.

Figure S4.2: Projected impacts under SSP2 when assuming linear (top, identical to Figure 5 in main text) and logarithmic (bottom) temperature increase.

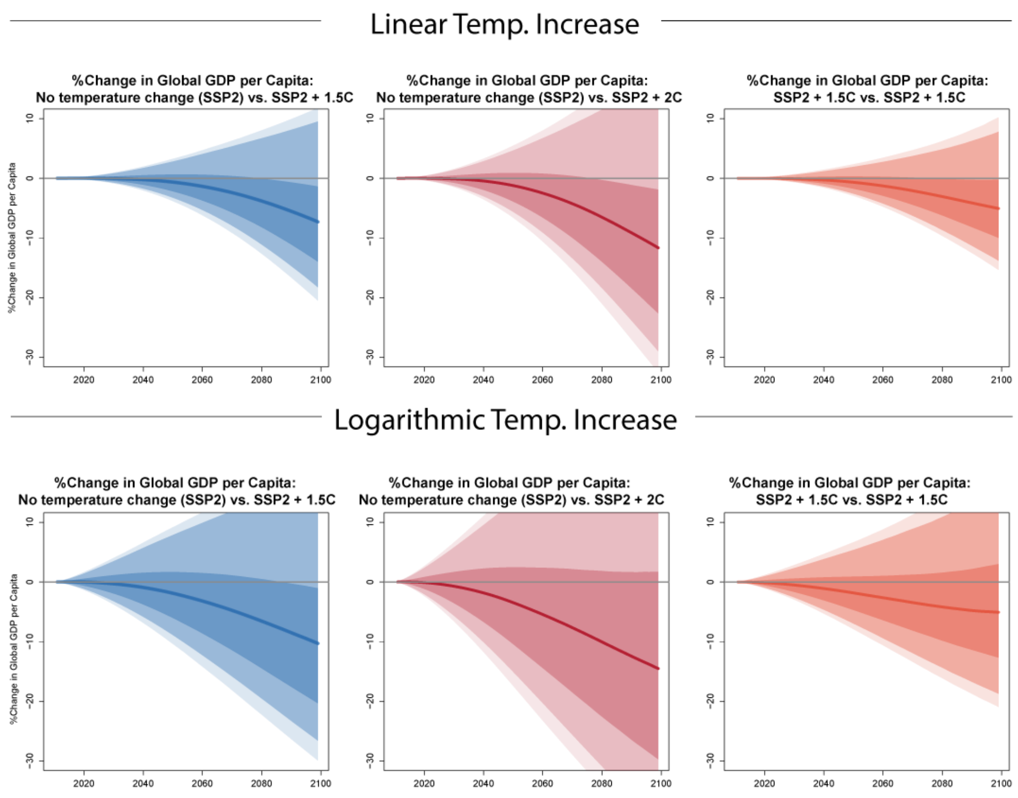

Supplementary Section S5: Mapping uncertainties around projected growth impacts

Figure 5 in the main text maps the projected growth rate impacts showing the magnitude of impacts for countries for which the projected 95% range of projected growth rate change falls outside of zero. This naturally imposes a sharp threshold (of 95%) on whether growth effects are labelled as significant or not. To map the range of uncertainty (as in Figure 4), here we show in Figure S5 the projected sign of the impact on growth rates (negative, or positive) using the 95% cut-off (as in Figure 5), as well as a 67% cut-off (approx. +-1SD in a normal distribution). Negative impacts (red and orange) are concentrated around the Tropics, while there a few positive projected impacts in the higher latitudes of the Northern Hemisphere.

Figure S5: Uncertainties around projected growth impacts. Maps show the sign of projected growth impacts, where projected negative growth effects are shown in red (if the 95% range falls below of zero) and orange (if the 67% range falls below zero). Positive growth effects are shown in dark (for 95%) and light (for 67%) blue.

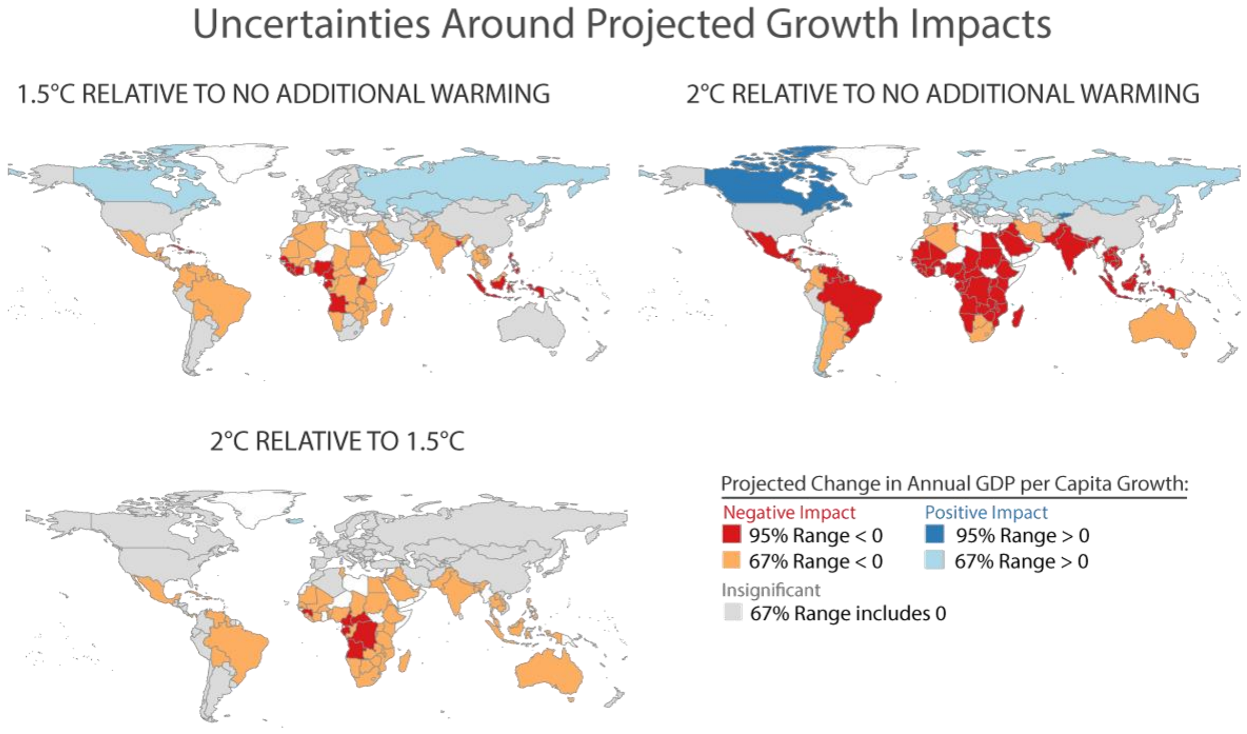

Supplementary Section S6: Alternative Construction of Within-Year Climate Variability

Section 4 in the main text reports model results using within-year variability of temperature and precipitation constructed as the variance of monthly temperatures and precipitation at the country level. This means that measures of within-year variability are averaged over large spatial areas. An alternative way to measure country-level within-year variation is to calculate the variation at the grid-cell level prior to aggregation to country level. Figure S6 plots the variance of monthly mean temperatures and precipitation constructed at the gridcell-level against the variance calculated at the country-level. There is little difference between cell and country-level temperatures, while precipitation variability is higher at the cell level. We report regression results with variability constructed at the grid-cell level in Table S6.1. The overall results are unaffected and near-identical to those using country-level variability (Table 1).

Figure S6: Variability of temperatures (left, red) and precipitation (right, blue) calculated on the grid-cell level (y-axis) plotted against variability calculated after aggregating to a country average (x-axis). The 45-degree line is shown in gray.

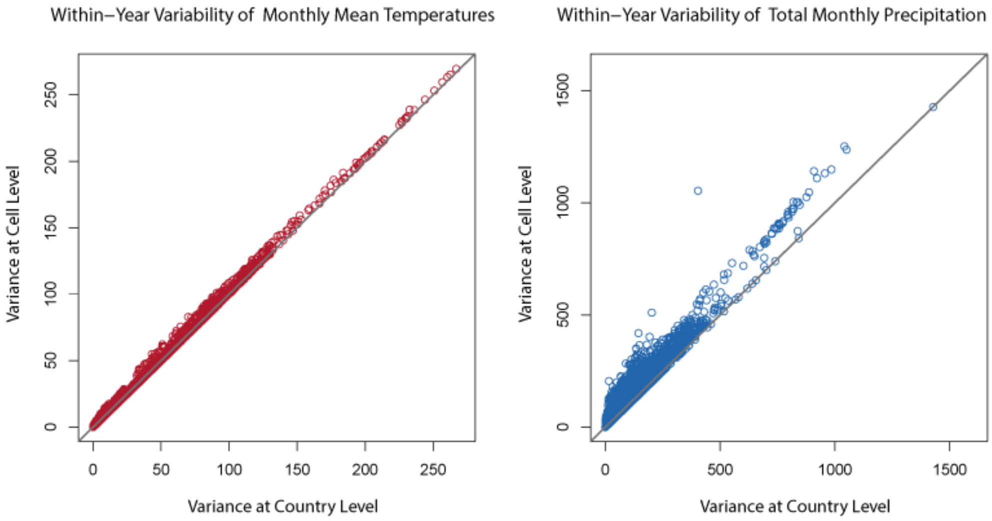

Table S6.1: Estimation Results from Equation [1] using within-year variability constructed on grid-cell level prior to aggregation to the country level. Estimated models M1.2 and M4.2 are identical to M1 and M4 except for the Var(Temp) and Var(Precip) and reported here without IIS.

Dep.Variable:	$y_{i,t}=\Delta\ln(GDPpc)_{i,t}$	$y_{it}=\Delta\%GDPAgric_{i,t}$
	M1.2: Within Yr. + Grid-Var	M4.2: Within Yr. + Grid-Var
$y_{i,t-1}$	0.1243 (0.0121)***	-0.2709 (0.0142)***
Temp.	0.0165 (0.0039)***	-0.0017 (0.0083)
Temp. Squared	-0.0005 (0.0001)***	-0.0004 (0.0002)
Precip.	0.0015 (0.0013)	0.0103 (0.0025)***
Precip. Squared	-0.0001 (0.0001)	-0.0003 (0.0001)***
Var(Temp.) (Grid-cell level)	0.0002 (0.0002)	-0.0022 (0.0004)***
Max(Temp.)	-0.0007 (0.0013)	0.0022 (0.0025)
Min(Temp.)	-0.0008 (0.0009)	-0.0038 (0.0018)*
Var(Precip.) (Grid-cell level)	-0.00006 (0.00003)	<0.0001 (0.0001)
Max(Precip.)	0.0004 (0.0002)	-0.0004 (0.0005)
Min(Precip.)	<0.0001 (0.0007)	0.0001 (0.0013)
Observations	7007	5034
Log Lik.	10809.10	5385.25

Standard errors given in parentheses. Significance: * 5%, **1%, ***0.1%..

References in the Supplementary Material

Burke M, Hsiang S, Miguel E. 2015 Global non-linear effect of temperature on economic production. Nature 527, 235–239. (doi:10.1038/nature15725)

IIASA. 2016 SSP Database, 2012–2016. See https://tntcat.iiasa.ac.at/SspDb.

Karl, T., Nicholls, N. and Ghazi, A. (1999). CLIVAR/GCOS/WMO Workshop on Indices and Indicators for Climate Extremes Workshop Summary. *Weather and Climate Extremes*, pp.3-7.

Mitchell D et al. 2017 Half a degree additional warming, prognosis and projected impacts (HAPPI): background and experimental design. Geosci. Model Dev. 10, 571–583. (doi:10.5194/gmd-10-571-2017)
